# Production of Transgenic-Cloned Pigs Expressing Large Quantities of Recombinant Human Lysozyme in Milk

**DOI:** 10.1371/journal.pone.0123551

**Published:** 2015-05-08

**Authors:** Dan Lu, Shen Liu, Shengzhe Shang, Fangfang Wu, Xiao Wen, Zhiyuan Li, Yan Li, Xiaoxiang Hu, Yaofeng Zhao, Qiuyan Li, Ning Li

**Affiliations:** 1 The State Key Laboratory for Agro-biotechnology, China Agricultural University, Beijing, China; 2 College of Animal Science and Technology, Yunnan Agricultural University, Kunming, China; 3 Beijing Genfucare Biotechnology Company, Beijing, China; Institute of Farm Animal Genetics, GERMANY

## Abstract

Human lysozyme is a natural non-specific immune factor in human milk that plays an important role in the defense of breastfed infants against pathogen infection. Although lysozyme is abundant in human milk, there is only trace quantities in pig milk. Here, we successfully generated transgenic cloned pigs with the expression vector pBAC-hLF-hLZ-Neo and their first generation hybrids (F1). The highest concentration of recombinant human lysozyme (rhLZ) with in vitro bioactivity was 2759.6 ± 265.0 mg/L in the milk of F0 sows. Compared with wild-type milk, rhLZ milk inhibited growth of *Escherichia coli* K88 during the exponential growth phase. Moreover, rhLZ in milk from transgenic sows was directly absorbed by the intestine of piglets with no observable anaphylactic reaction. Our strategy may provide a powerful tool for large-scale production of this important human protein in pigs to improve resistance to pathogen infection.

## Introduction

Lysozyme is a natural, non-specific, immune factor, which widely exists in animals, plants, and microorganisms. Lysozyme has broad-spectrum antimicrobial activities both *in vivo* and *in vitro* against Gram-positive and-negative bacterial species, including *Bacillus subtilis*, *Bacillus cereus*, *Staphylococcus aureus*, *Escherichia coli*, *Klebsiella pneumoniae*, *Pseudomonas aeruginosa*, *Streptococcus agalactiae*, and *Salmonella typhimurium*, as well as the fungus *Candida albicans* [1–4]. Lysozyme directly kills bacteria (especially Gram-positive species) through hydrolysis of tetrasaccharide β-(1→4)-glycosidic linkages in the cell wall [5]. Besides muramidase enzymatic activity, previous studies have indicated that lysozyme may also possess a muramidase-independent mechanism to kill bacteria [1,6,7]. Since it received a status of “generally recognized as safe” by the World Health Organization and US Food and Drug Administration, lysozyme is now used widely as a food preservative [8]. Previous experimental and clinical studies demonstrated that lysozyme can enhance the function and proliferation of polymorphonuclear neutrophils and phagocytes by non-specific immune regulation, as well as plays an important role in antitumor activities [9–11]. Moreover, lysozyme can serve as a potential alternative to antibiotics, since it has demonstrated similar functions in improving growth performance of poultry and pigs [12–14].

During the first 12 weeks of the lactation period, lysozyme concentrations in human milk range from 0.27 to 0.89 g/L [15], that is 1500–4000 times greater than that present in the milk of cows, goats, and pigs [16,17]. Besides the high concentration in milk, human lysozyme (hLZ) also possesses much greater enzymatic activity and stability than that in other species [18]. Therefore, hLZ is more applicable in genetically modified animals. Studies of recombinant human lysozyme from transgenic goats demonstrated that it can reduce concentrations of detrimental microbes and enrich those of beneficial microbes, without disturbing overall community populations [19,20]. It also benefits gut morphology by increasing intestine villus height and thinning lamina propria [21].

Diarrheal diseases are problematic in intensive pig farms, as millions of piglets die annually because of bacterial and viral infections. By feeding pigs with rhLZ milk after infection with enterotoxigenic *E*. *coli*, diarrheal diseases can be overcome more rapidly. In fact, pigs fed rhLZ showed less intestinal inflammation, less damage to the intestinal villus, and faster recovery to normal proportions of blood leukocytes [21,22]. Together, these results demonstrated that rhLZ is a promising candidate for treatment of diarrhea among suckling pigs. Therefore, we attempted to produce genetically modified pigs expressing rhLZ in the milk to lay a foundation for breeding of diarrhea-resistant pigs.

Although large quantities of rhLZ can be produced by transgenic mice, goats, and cows that exceed 1.0 g/L [23–25], rhLZ expression in the transgenic pigs mammary glands remains less than satisfactory [17,26]. Previously, we produced rhLZ transgenic-cloned pigs with the vector pBC1-hLZ-GFP-Neo that resulted in an average rhLZ concentration in transgenic milk of 116.34 μg/mL. Piglets nursed by those transgenic pigs showed greater growth inhibition of *E*. *coli* in the duodenum and positive influence in intestinal morphology compared with piglets from control group [26]. However, we observed no other obvious improvements in piglets fed with transgenic milk because the low rhLZ concentration in milk might have been insufficient to greatly effect piglet health. Our previous study showed the vector pBAC-hLF-hLZ-Neo efficiently expressed rhLZ in the mammary gland of mice [24]. Thus, in the present study, we used this vector to produce transgenic pigs expressing high levels of recombinant human lysozyme in milk.

## Materials and Methods

### Ethics statement

Our study protocols were approved by the Institutional Animal Care and Use Committee of the China Agricultural University with approved number SKLAB-2012-04-05. All the procedures were performed in strict accordance with the *Guide for the Care and Use of Laboratory Animals*. We performed all surgeries under sodium pentobarbital anesthesia and tried our best to minimize animals suffering.

### Production of transgenic-cloned pigs

**Fig 1:**
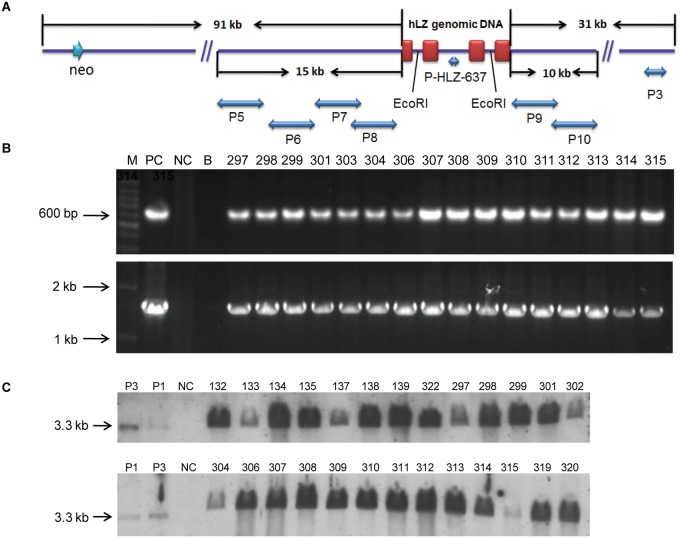
Production and identification of transgenic pigs. (A) Structure of expression vector pBAC-hLF-hLZ-Neo. It contains a 4.8-kb *hLZ* genomic DNA, 5’ and 3’ flanking region of the hLF gene and a selection cassette Neo. The lower bars represent the position of the PCR products from the primer pairs P5–P10, which were used for verification of the intactness of the BAC vector.(B) Identification of transgenic pigs by PCR analysis. PC, positive control vector; NC, genomic DNA from three WT pigs; B, water; lane 5–20: genomic DNA from transgenic cloned pigs; M, 100-bp DNA ladder or 1-kb DNA ladder.(C) Identification of transgenic pigs by southern blot analysis. P1, plasmid vectors with one copy of the hLZ gene; P3, plasmid vectors with three copies of the hLZ gene; NC, genomic DNA from a WT pig.

The expression vector pBAC-hLF-hLZ-Neo (Fig 1A), which was shown to express high levels of rhLZ in the mammary gland of transgenic mice [24], was used to produce transgenic-cloned pigs. Landrace fetal pig fibroblast lines were established and cultured as described previously [27]. The bacterial artificial chromosome (BAC) DNA was purified using the NucleoBond BAC 100 Kit (Macherey-Nagel GmbH & Co. KG, Duren, Germany) and the linearized pBAC-hLF-hLZ-Neo, containing *Not*I restriction sites, was introduced into porcine fetal fibroblasts using the Amaxa Nucleofector Transfection System (Amaxa Biosystems, Cologne, Germany). The nucleofector solution was from the Basic Nucleofector Kit for Primary Mammalian Fibroblasts (Lonza, Basel, Switzerland). Briefly, 5×10^6^ cells in 100 μL of Amaxa nucleofector solution were mixed with 3 μg of BAC DNA and immediately transferred to the Amaxa cuvette with a 2.5-mm gap using the preprogrammed settings T016. After 24 h of transfection, aminoglycoside antibiotic G418 was added into the culture at a final concentration of 600 μg/mL and the cells were cultured under selection for two weeks. The surviving cells were then passaged twice under the selection of G418 at a final concentration of 300 μg/mL. At the end of culture, the cells were frozen and stored in liquid nitrogen. Nuclear transfer was performed as described previously [26,28,29].

### Identification of transgenic-cloned pigs by PCR and Southern blot analysis

Tissues from ears of transgenic-cloned pigs and wild-type (WT) pigs were used to extracted genomic DNA, which then subjected to PCR and southern blot analysis using the primers listed in S1 Table. Water and genomic DNA from WT pigs were used as PCR controls. Primers P-HLZ-637 and P3 were used to identify the positive transgenic-cloned pigs, and their product sizes were 637 bp and 1.5 kb, respectively. For southern blot analysis, 10 μg of genomic DNA from transgenic-cloned pigs and WT pigs was digested with *Eco*RI. After resolved by 0.8% agarose gel electrophoresis and transferred to nylon membrane (Roche Applied Science, Mannheim, Germany), the samples were hybridized with a digoxigenin (DIG)-labeled probe amplified with the primer P-HLZ-637 to produce a 3.3-kb positive hybridization signal.

### RT-PCR analysis of rhLZ mRNA

We extracted total RNA from different tissues using TRIzol reagent (Tiangen Biotech (Beijing) Co., Ltd., Beijing, China). First-strand cDNA of *hLZ* and *GAPDH* (internal control) was synthesized using oligo-dT primers. RT-PCR primers were designed based on the *hLZ* coding sequences (S1 Table).

### Copy number detection by qPCR

The copy number of transgene was detected by qPCR, as described previously [30], using Roche LightCycler 480 System (F. Hoffmann-La Roche AG, Basel, Switzerland). First, all the genomic DNA from transgenic pigs and WT pigs were diluted to 10 ng/μL. To establish a standard curve, the plasmid DNA with different transgene copies (1, 2, 4, 8, 16 and 32 copies) were mixed with WT pigs genomic DNA. All the reactions were performed in 20-μL reaction valume contained template DNA (1 μL), primers (3 μL), Power SYBR Green Mix (10 μL) and ddH_2_O (8.4 μL). We used myostain gene (*MSTN*; gene ID, 399534) as an internal control to calculate transgene copy number. The qPCR primers were designed based on the *hLZ* and *MSTN* coding sequences (S1 Table).

### Milk sample collection

Milk samples were collected from transgenic-cloned sows at different time points (3 h, 6 h, 9 h, 12 h, 24 h, 48h, 7 d, 14 d, 21d, and 28 d) after the completion of farrowing. After aliquoted, all samples were stored at -20°C.

### Blood sample collection and histamine analysis by ELISA

Two groups of WT piglets were nursed by transgenic-cloned sows and WT sows for 21 days and their blood samples were collected once a week. The serum samples were obtained by centrifuging the blood samples at 3000 × g for 10 min and stored at -80°C. Histamine concentrations in blood samples were measured using an ELISA kit (Beijing Dong Songs biological technology Co., Ltd., Beijing, China) with a sensitivity detection threshold of 0.3–16 μg/L. After stopping the reaction, absorbance at a wavelength of 450 nm was measured by a SpectraMax 340 PC Plate Reader (Molecular Devices Corp., Sunnyvale, CA, USA).

### Western blot analysis

All milk samples were centrifuged at 10,000 × g for 15 min at 4°C for defatting. After diluted three times with ddH_2_O, 3 μL samples were resolved on a 15% SDS–PAGE gel and transferred to a nitrocellulose membrane. The primary antibody was polyclonal rabbit anti-hLZ (dilution, 1:2,000; US Biological Inc., Swampscott, MA, USA) and the secondary antibody was horseradish peroxidase-conjugated goat anti-rabbit IgG (dilution, 1:20,000; Sino-American Co., Beijing, China).

### rhLZ quantification using a radioimmunoassay (RIA)

For the RIA, hLZ (Sigma-Aldrich Corporation, St. Louis, MO, USA) was radiolabeled by the chloramine-T method as described previously [31]. The ^125^I-labeled samples were purified by chromatography using Sephadex G-25 column. All milk samples were diluted 100-fold. The five hLZ standards ranged from 0.1 to 1,000 μg/mL. Diluted samples or standard protein solutions were incubated for 16–24 h in 100 μL 16,000 cpm of ^125^I-labeled hLZ and a polyclonal rabbit anti-hLZ antibody (dilution, 1:100). Then donkey anti-rabbit immune precipitating reagent were added for an additional 15 min incubation. Bound and free ligands were separated by centrifugation at 3500 rpm/min for 15 min. Radioactivity was measured using an automatic gamma counter (Xi'an Nuclear Instrument Factory, Xi'an, China).

### Lysozyme activity assays

We used both a turbidimetric assay and gel diffusion assay to measure the enzymatic activity of rhLZ in milk. As we have been described previously [26,32], *M*. *lysodeikticus* (China General Microbiological Culture Collection Center, Beijing) is an sensative substrate of lysozyme, since the cell wall of this bacteria is composed of peptidoglycan polymer. For the turbidimetric assay, we prepared *M*. *lysodeikticus* cell suspension with potassium phosphate buffer, which the absorbance of 450 nm (A450) is around 0.7. Diluted milk samples (100 μL) from trasgenic pigs were added into 2.5 mL of *M*. *lysodeikticus* cell suspension and monitor the reduction of A450. One unit will produce a ΔA450 of 0.001 nm/min and all samples were measured three times. For the gel diffusion assay, we prepared the agar plate containing *M*. *lysodeikticus* first, and put the 6-mm filter paper discs on those plate. Diluted milk samples (dilution, 1:3, 6 μL) were added to 6-mm quantitative filters. The positive control is commercial natural hLZ standard (1 μg) and the negative contol is milk from WT pig.

### Bacterial strains and culture

*E*. *coli* K88 is a Gram-negative bacteria that is the main cause of diarrhea in pigs, and previous study described the use of *E*. *coli* K88 to induce diarrhea in pigs [33]. Therefore, we choose this pathogen to detect the influence of rhLZ in milk on the growth of piglets. *Escherichia coli* K88 (O149:K91, K88ac) was purchased from the China Veterinary Culture Collection Center. The bacteria were grown in Luria-Bertani medium at 37°C for 12 h and then diluted to approximately 2000 CFU/mL. The experimental groups were fed with defatted milk from transgenic pigs with final rhLZ concentrations of either 100 or 50 μg/mL, while the control group was fed the same volume of defatted WT milk. For each group, 1 mL of diluted bacteria was added to the culture tubes for a final volume of 8 mL. We set up two replicates for each group. All groups were cultured at 37°C for 3 h and then the concentration at an optical density at 600 nm (OD600) was measured using a spectrophotometer (Molecular Devices Corp., Sunnyvale, CA, USA). Afterward, the OD600 value was measured every hour for 5–7 h. All experimental data was analyzed by a Student’s *t*-test using SPSS software (ver. 19.0; SPSS Inc., Chicago, IL, USA). The results of comparisons with P<0.05 and P<0.01 were considered statistically significant and very statistically significant, respectively.

## Results

### Production and identification of transgenic-cloned pigs

The expression vector pBAC-hLF-hLZ-Neo was constructed previously by replacing the human lactoferrin (*hLF*) gene with a 4.8-kb *hLZ* genomic DNA fragment in hLF BAC, and it also contained a Neo cassette for selection (Fig 1A). This vector was used to generate transgenic mice with large quantities of rhLZ expressed in milk [24]. After cell selection, we retrieved five positive cell colonies for use in somatic cell nuclear transfer. We transferred 3338 embryos into eight recipient gilts (Table 1), resulting in the birth of 45 female cloned pigs, although four died soon after birth. The integration of the *hLZ* gene was confirmed by PCR analysis. The primers P-HLZ-637 and P3 were used to amplify positive amplicons with expected sizes of 637 bp and 1.5 kb, respectively. The results showed that 31 pigs were transgene positive (Fig 1B). The integration of *hLZ* in transgenic pigs was also confirmed by southern blot analysis (Fig 1C). The integrity of transgenic constructs in all transgenic-cloned pigs were detected by long-fragment PCR. Five pairs of primers, P5–P10 (S1 Table), that produced amplicons with partial overlaps were used to amplify the 15-kb 5’ flanking region and 10-kb 3’ flanking region of the *hLF* gene (Fig 1A). We detected these regional regulatory fragments in all piglets. Copy numbers of the *hLZ* gene in all transgenic pigs were also detected by qPCR. Pigs from the same cell colonies showed similar transgene copy numbers. Among them, pigs from the cell colonies Slw12-lf-LYZ3-2 and Slw12-lf-LYZ25 had up to seven copy numbers (Table 2).

**Table 1:** Transplantation results of transgenic cloned embryos.

Donor cell colonies	Recipient gilts	Number of transferred embryos	Viable offspring	Transgenic offspring
Slw12-lf-LYZ3	1431	380	7	8
Slw12-lf-LYZ3	1281	400	10	1
Slw12-lf-LYZ25	235	420	3	3
Slw12-lf-LYZ25	468	400	8	8
Slw12-lf-LYZ25	880	380	7	6
Slw12-lf-LYZ11	344	390	3	2
Slw12-lf-LYZ11	702	590	1	1
Slw12-lf-LYZ12	463	378	2	2

**Table 2:** Expression level of rhLZ in the milk of transgenic pigs.

NO.	Cell clone	Copy number	Average expression level (mg/L)*	Expression level for each copy (mg/L)
133	Slw12-lf-LYZ3-1	1	508.4 ± 153.9	508.4
137	Slw12-lf-LYZ3-1	1	600.5 ± 125.4	600.5
132	Slw12-lf-LYZ3-2	6	1625.5 ± 283.3	270.9
138	Slw12-lf-LYZ3-2	7	1852.2 ± 618.8	264.6
303	Slw12-lf-LYZ11	1	552.5 ± 210.7	552.5
315	Slw12-lf-LYZ12	3	1284.2 ± 450.3	428.1
299	Slw12-lf-LYZ25	7	1450.5 ± 344.8	207.2
301	Slw12-lf-LYZ25	7	1491.3 ± 337.7	213.0
312	Slw12-lf-LYZ25	7	1557.0 ± 402.2	222.4
313	Slw12-lf-LYZ25	7	1108.7 ± 378.2	158.4

* Data are presented as averages ± standard deviations.

### rhLZ expression

**Fig 2:**
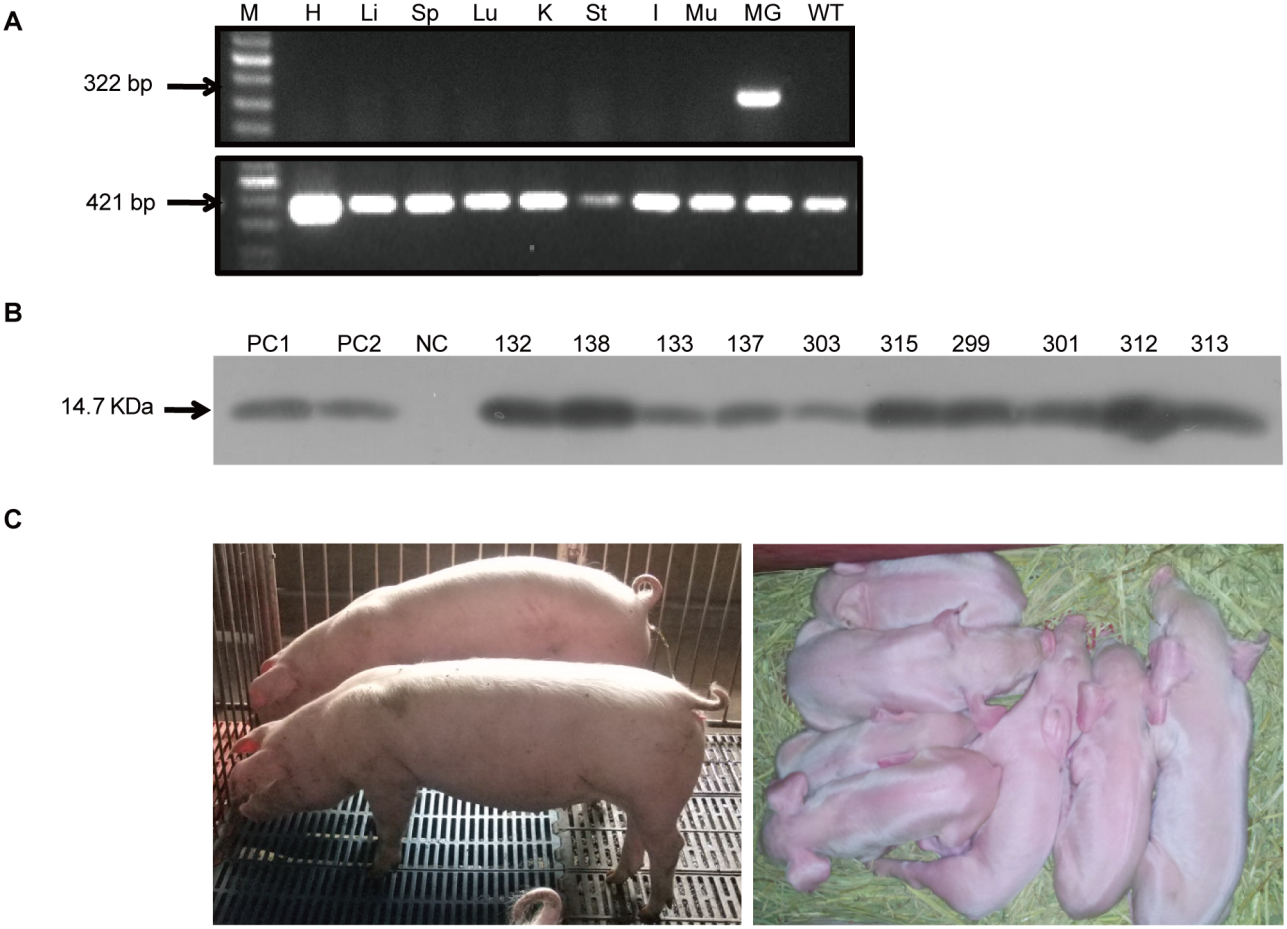
rhLZ expression in transgenic-cloned pigs. (A) RT-PCR analysis of RNA extracted from the mammary gland and other tissues of transgenic-cloned pigs. The sizes of RT-PCR products from rhLZ and GAPDH were 322-bp and 421-bp, respectively. M, 1-kb DNA ladder; WT, mammary gland tissue from wild-type pig; H, Li, Sp, Lu, K, St, I, Mu, and MG represent heart, liver, spleen, lung, kidney, stomach, intestine, muscle and mammary gland from transgenic pig, respectively.(B) Western blot analysis of rhLZ expression in transgenic pigs. Milk samples from transgenic-cloned sows on lactation day 1 were collected and detected by western blot analysis. PC1, 100 ng commercial natural hLZ standard; PC2, 50 ng commercial natural hLZ standard; NC, milk from WT pigs; lanes 4–13, diluted milk from transgenic-cloned pigs (dilution, 1:3, 3 μL).(C) Image of F0 and F1 transgenic pigs. Left, 4-month-old F0 transgenic pigs; Right, F1 neonatal transgenic piglets.

RT-PCR was conducted to assess *rhLZ* expression in transgenic-cloned pigs. Briefly, we isolated total RNA from different tissues, including lactating mammary gland, liver, heart, spleen, kidney, stomach, lung, intestine, and muscle of transgenic sows. As a negative control, the total RNA from the mammary gland tissue of a WT sow was also isolated. To make sure extraction of RNA and PCR had been done properly, the housekeeping gene *GAPDH* was used as an internal control. As expected, we detected the *rhLZ* expression only existed in the mammary gland tissues from the transgenic sows (Fig 2A). To detect rhLZ expression in milk, 10 transgenic pigs were mated with a WT Landrace boar as the founder animals. We collected milk samples from the 10 transgenic sows at 24 h after farrowing. The result of western blot showed that all the samples from transgenic sows contained rhLZ, and the molecular weight is the same as the natural hLZ standard (14.7 kDa) (Fig 2B).

### rhLZ concentration and activity

**Fig 3:**
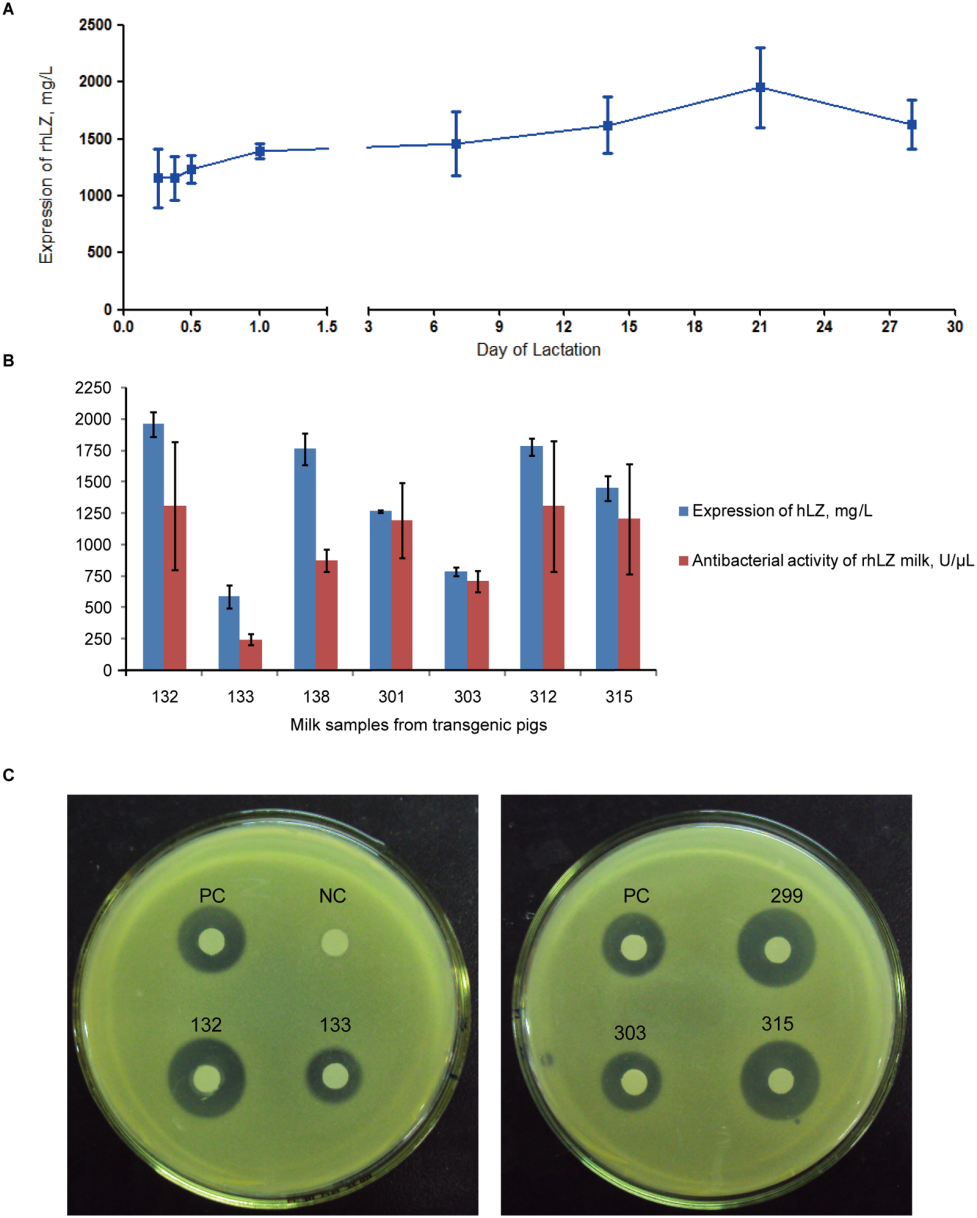
rhLZ concentration and enzymatic activity in transgenic milk. A)hLZ expression levels of transgenic sows during the lactation period. Values are means ± standard deviations. B)Expression level and antibacterial activity of rhLZ on day 7 in transgenic milk. Values are means ± standard deviations. C)The gel diffusion assay. Dilution milk samples (dilution, 1:3, 6 μL) were added to 6-mm small white quantitative filters. PC, commercial natural hLZ standard (1 μg); NC, milk from WT pig.

We assessed rhLZ content in all milk samples using a RIA. As shown in S2 Table, rhLZ expression varied in different transgenic pigs and at different stages of lactation. The concentrations of rhLZ on all assessed time points of lactation ranged from 342.0 to 2759.6 mg/L and the highest concentration was present at 48 h postpartum from transgenic pig no. 138 (S2 Table). We compared the rhLZ expression levels in different transgenic pigs with different copy numbers. The results showed that higher copy number resulted in higher concentration of rhLZ. But this relationship was not linear. The one copy transgene transgenic pigs (133, 137 and 303) showed higher rhLZ concentration per transgene copy that is above 500 mg/L. However, transgenic pigs with 7 transgene copies showed a lower rhLZ concentration per transgene copy that is around 200 mg/L (Table 2). Next, we attempted to identify trends in rhLZ expression in milk during lactation with four transgenic pigs from the same clone (Slw12-lf-LYZ25) (Fig 3A). The average expression level of rhLZ gradually increased from lactation day 3 to day 21 compared to that of colostrum. On lactation day 28, the average rhLZ concentration slightly decreased.

Turbidimetric assay was used to quantify rhLZ enzymatic activity. Here we examined the milk samples from transgenic sows on lactation day 7. As shown in S2 Table, rhLZ activity ranged from 246.0 ± 44.2 to 1311.0 ± 508.6 U/μL. The general trend of rhLZ expression level and enzymatic activity at the indicated time points was similar (Fig 3B). Gel diffusion assay was also used as a more intuitive detection method to observe the enzymatic activity of transgenic milk. The negative controls were water and milk from WT sow and the positive control was 1.0 μg commercial natural hLZ standard (Fig 3C). From the transparent zones around filters, we can see that rhLZ activity from sample nos. 133 and 303 was quite lower than the activity of positive control, while that from sample nos. 132, 299, and 315 was much higher than demonstrated by the positive control. These results were consistent with those of western blotting and RIA analysis.

### rhLZ milk can inhibit growth of *E*. *coli* K88 *in vitro*

**Fig 4:**
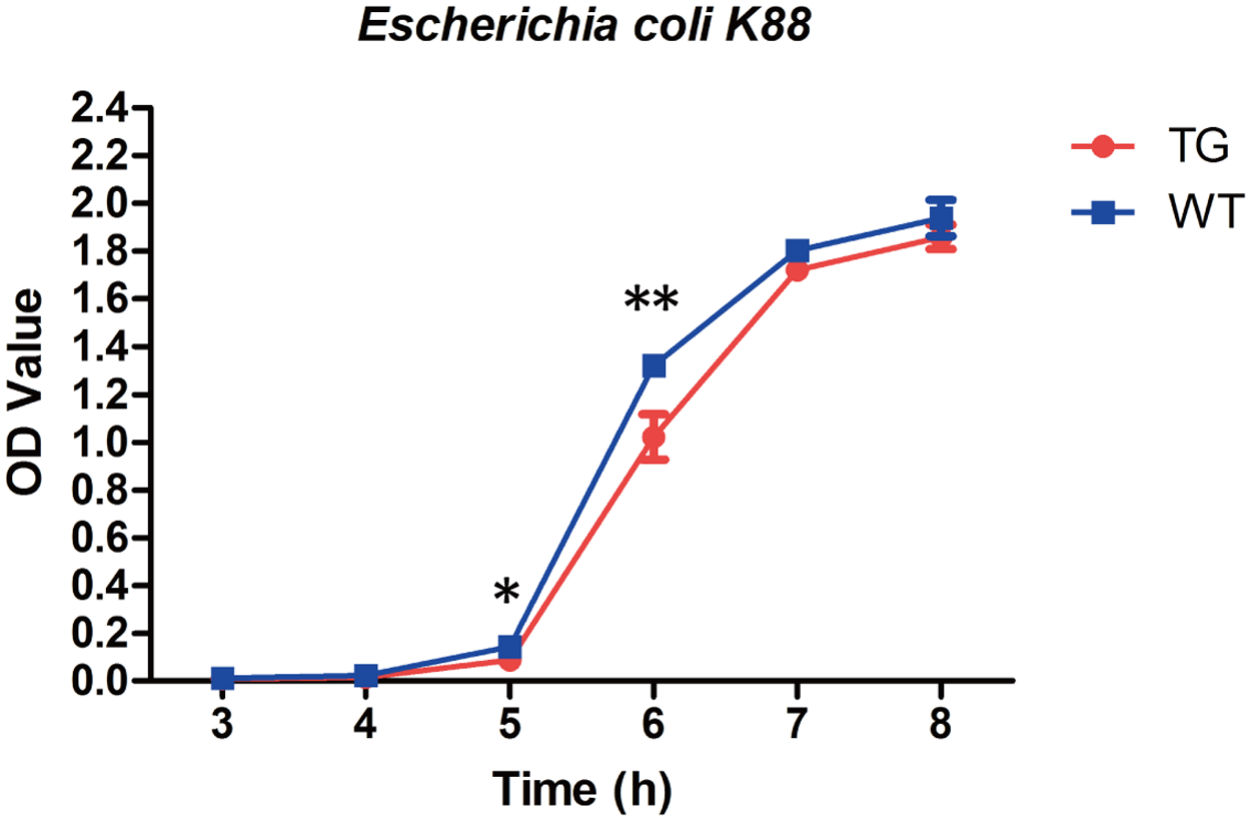
The influence of rhLZ milk on growth of *E*. *coli* K88. The influence of rhLZ transgenic milk on the growth of *E*. *coli* K88. Values are showed as means ± standard deviations. The growth of individual bacterial strains in the presence of milk from transgenic pigs was significantly different from controls (*p < 0.05, **p < 0.01).

To detect the influence of rhLZ in milk on the growth of *E*. *coli* K88, the *E*. *coli* K88 was incubated with rhLZ milk and observed by measuring OD600 value. With a concentration of rhLZ in milk of 50 mg/L, there was no obvious growth inhibition of *E*. *coli* K88 at different time points (S3 Table). When the concentration of rhLZ in milk was increased to 100 mg/L, the growth of *E*. *coli* K88 showed significant differences at 5 and 6 h compared with the control group (*p* = 0.0004 and 0.0146, respectively; S3 Table). The OD values of samples from the rhLZ milk-treated group were much lower than those of the control group. Those results indicated that the presence of rhLZ can inhibit the growth of *E*. *coli* K88 during the exponential growth period (Fig 4 and S3 Table).

### Absorption of rhLZ in piglets

**Fig 5:**
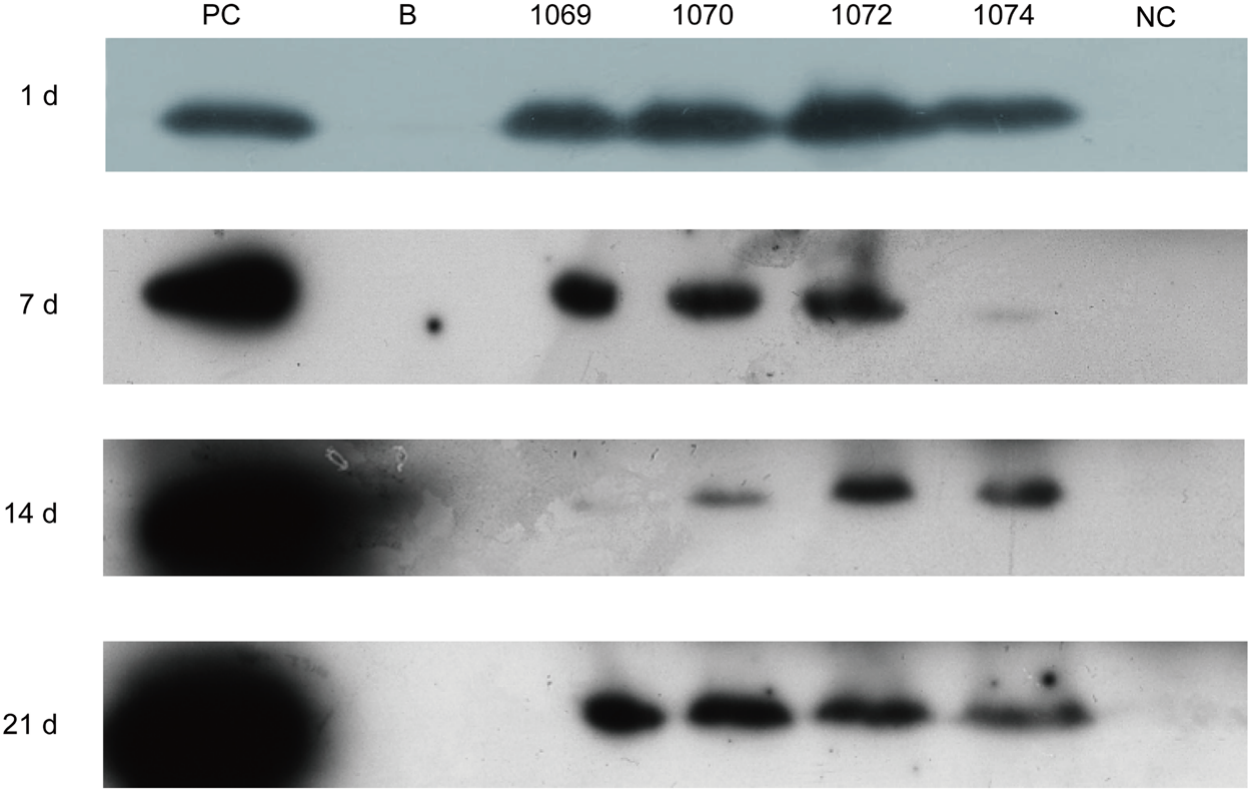
Detection of rhLZ in plasma of piglets. Blood samples were detected by western blot analysis. PC, commercial natural hLZ (1 μg); NC, blood from piglets nursed by WT sows; lanes 3–6, diluted blood samples (5 μL) from piglets nursed by transgenic sows.

To study the absorption of rhLZ in piglets, blood samples from suckling piglets nursed by transgenic-cloned sows and WT sows were collected on postpartum days 1, 7, 14, and 21. Western blot analysis was conducted to identify the presence of rhLZ during the lactation period. Commercial hLZ (1 μg) as a positive control and 5 μL of blood samples were loaded into 15% gel for SDS-PAGE. The results demonstrated positive bands of samples from piglets nursed by transgenic sows, while there were no bands of samples collected from piglets nursed by non-transgenic sows. Moreover, rhLZ was absorbed by nursing piglets during the entire lactation period. From the band signals and time of exposure, the concentration of rhLZ in piglets decreased from day 1 to 21 (Fig 5).

**Fig 6:**
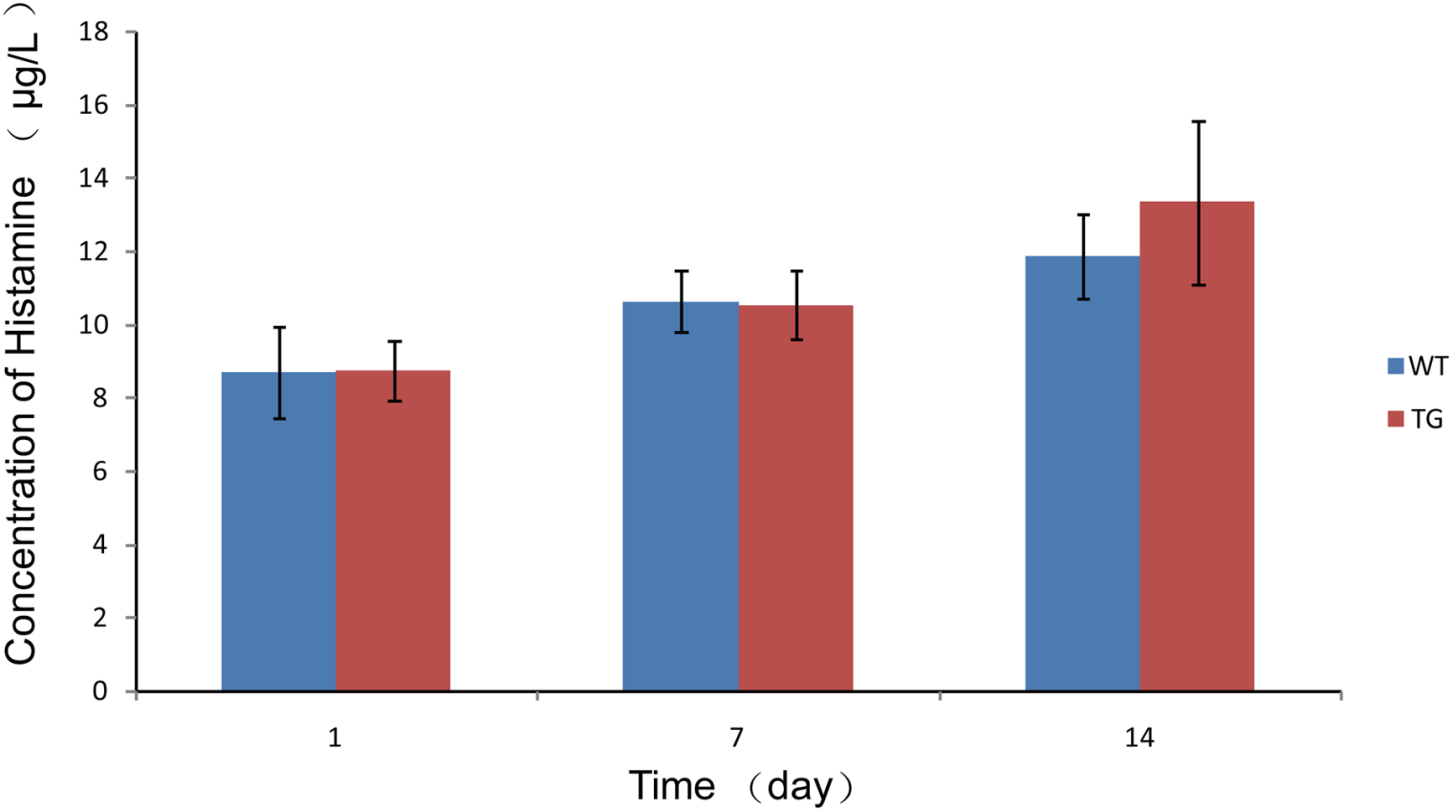
Detection of histamine in plasma of piglets. Data are means ± standard deviations. There was no significant difference in the histamine concentrations in the blood of piglets nursed by transgenic-cloned and WT sows (p > 0.05).

We next determined whether a high concentration rhLZ as a foreign protein in piglet blood could cause an immunoreaction, such as a hypersensitivity response. As shown in Fig 6, there was no significant difference in the histamine concentrations in the blood of piglets nursed by transgenic-cloned and WT sows (*p* > 0.05).

## Discussion

hLZ is a major component in human milk and plays an important role in the innate immune response of breastfed infants against infection of pathogenic bacteria and viruses. Here, we produced a herd of rhLZ transgenic cloned pigs with high rhLZ expression levels in milk. To the best of our knowledge, this is also the highest expression level of rhLZ reported in genetically modified pigs. Since rhLZ can improve the gut health of pigs and resist diarrhea, our model is ideal to explore whether pigs can achieve resistance to diarrheal diseases through genetic modification.

Recently, the use of yeast artificial chromosomes (YACs) and BACs to express foreign proteins has become more prevalent, in which transgenes can achieve expression at physiological levels. High expression levels of heterologous proteins, such as human and goat alpha-lactalbumin [34], porcine whey acidic protein [35], porcine follicle-stimulating hormone [36], human lactoferricin [37], and hLZ [24], have been achieved with the use of both YACs or BACs. Previous studies demonstrated that transgene expression driven by BAC demonstrated copy number-dependent and position-independent [35]. From our experiment results, the higher copy number resulted higher concentration of rhLZ in milk, but rhLZ yield per transgene copy changed significantly in different trasgenic pigs. The estimated rhLZ yield per transgene copy varied from 158.4 to 600.5 mg/L among the 10 transgenic pigs. These findings indicated that the positional effect in our study may play a more important role in the expression of rhLZ in transgenic pigs.

During the lactation period, the presence of rhLZ was continuous in the milk of transgenic sows. As reported in previous studies of human milk, hLF expression was highest in colostrum (5–7 g/L) and decreased over time with the period of lactation (3.7 g/L in transitional milk and 1–2 g/L in mature milk) [38–40]. However, although all the regulatory region of vector pBAC-hLF-hLZ-Neo was from the hLF gene, the expression pattern of rhLZ was different from that of hLF. On the contrary, it was more similar to the expression pattern of hLZ, which showed a trend of gradual increase during the lactation period [15].

Previous studies have indicated that goat milk with rhLZ can slow the growth of *E*. *coli* [3] and *E*. *coli* is the main bacterial source of infection inducing diarrhea in piglets. In our study, we found similar patterns of growth inhibition of *E*. *coli* K88, which occurred during the early stage of logarithmic growth. Besides, in vivo experiments confirmed that lysozyme can help pigs to recover from diarrhea after infection with *E*. *coli* [21]. These results paved a good foundation to support our strategy of breeding diarrhea-resistant pigs by genetically adding rhLZ in milk.

It is essential that piglets absorb intact rhLZ for proper function of the enzyme. Several studies indicated that the intestine can absorb intact lysozyme by endocytosis and paracellular pathways using immunological or biological methods, and the intestinal absorption of lysozyme is segment-selective, absorbing preferentially from the upper intestine [41–43]. In our study, intact rhLZ was detected by western blotting of plasma from piglets, which was consistent with the findings of previous reports [41,43]. The decrease in plasma rhLZ content may be due to maturation of the intestine with tightening the adjacent epithelial cells. Lysozyme is a major egg-white allergen that can induce food-dependent and exercise-induced anaphylaxis [44]. In the present study, although a large amount of rhLZ, as a heterologous protein, existed in the plasma of piglets, no anaphylaxis was detected by measurement of plasma histamine content.

In summary, we efficiently produced transgenic-cloned pigs with the expression vector pBAC-hLF-hLZ-Neo. The highest concentration of rhLZ presented in the transgenic sows was 2759.6 ± 265.0 mg/L, which demonstrated bioactivity in vitro. rhLZ was directly absorbed by the intestine of piglets through milk from transgenic sows and there were no anaphylactic reaction. Our strategy may provide a powerful tool for large-scale production of this important human protein in pigs to improve their resistance against diarrheal diseases.

## Supporting Information

S1 Table Primers for transgenic pigs verification. *All the primers were uesed to detected rhLZ mice and pigs in our previous studies[24,26].
(DOCX)

S2 Table Expression level of rhLZ in the milk of transgenic pigs (mg/L) and rhLZ enzymatic activities (U/μL) of milk collected on day 7. Values are averages ± standard deviations. Some samples at particular time were not collected, and here we use “-” to indicate.
(DOCX)

S3 Table The influence of rhLZ transgenic milk on the growth of *Escherichia coli K88*. Values are averages ± standard deviations.
(DOCX)
