# Supplementary material for: Production of Transgenic-Cloned Pigs Expressing Large Quantities of Recombinant Human Lysozyme in Milk
Source: PLoS One. 2015 May 8;10(5):e0123551. doi: 10.1371/journal.pone.0123551 (PMC4425539; doi:10.1371/journal.pone.0123551)
Supplement: S1 Table — *All the primers were uesed to detected rhLZ mice and pigs in our previous studies[24,26]. (DOCX) [file pone.0123551.s001.docx]

**S1 Table. Primers for transgenic pigs verification*.**

| Primer name | Primer sequence | Utility |
| --- | --- | --- |
| P3-F | 5’-TTCCTTCCACCACTGTTGAG-3’ | PCR detection of transgenic founders ( 1.5 kb) |
| P3-R | 5’-CAAATACCTCTGCCGCTGTT-3’ |  |
| P5-F | 5’-ACAATCCAGGGACATGATACAGA-3’ | Intactness of BAC verification (4.1 kb) |
| P5-R | 5’-GATGGGACTTTCCGTACAAT-3’ |  |
| P6-F | 5’-CTGTTCAACATTGTACGGAAAGT-3’ | Intactness of BAC verification (4.8 kb) |
| P6-R | 5’-ATGTCTATACGGGTTATGG-3’ |  |
| P7-F | 5’-CATAACCCGTATAGACATAGGTG-3’ | Intactness of BAC verification (4.4 kb) |
| P7-R | 5’-GGCCCTCTTAAGTATGTTACAG-3’ |  |
| P8-F | 5’-ACACATTGCCAGAGTAGAGTAGA-3’ | Intactness of BAC verification (3.7 kb) |
| P8-R | 5’-GCCTTGGTGTCTTAGTATTG-3’ |  |
| P9-F | 5’-ACAGAAGTTATTAGTGCGACCAA-3’ | Intactness of BAC verification (2.2 kb) |
| P9-R | 5’-GCTACACGGGAAATCTATCTATC-3’ |  |
| P10-F | 5’-GGTCTATTAGAGGCTATGGGTCG-3’ | Intactness of BAC verification (2.9 kb) |
| P10-R | 5’-GGCCCTCTTAACACTTCC-3’ |  |
| P-HLZ-637-F | 5’-TTATACACACGGCTTTAC-3’ | PCR digoxigenin-labeled probe synthesis (637 bp) |
| P-HLZ-637-R | 5’-CAGCATCAGCGATGTTATCT-3’ |  |
| P-hLZ-322F | 5’-ATCAGCCTAGCAAACTGGAT-3’ | RT-PCR for hLZ (322 bp) |
| P-hLZ-322F | 5’-CTCCACAACCTTGAACATAC-3’ |  |
| P-GAPDH-421F | 5’-ACCCAGAAGACTGTGGATGG-3’ | RT-PCR for GAPDH control (421 bp) |
| P-GAPDH-421R | 5’-CCCTGTTGCTGTAGCCAAAT-3’ |  |
| P-hLZ-112F | 5’-TGCTGGGTGCCTGAGATTCA-3’ | Q-PCR for hLZ copy number (112 bp) |
| P-hLZ-112R | 5’-AGTTCAAAATGGGAAATAACTGG-3’ |  |
| P-MSTN-110F | 5’-TCTGAGACCCGTCAAGACTCCTA-3’ | Q-PCR for MSTN control (110 bp) |
| P-MSTN-110R | 5’-TGTCAAGTTTCAGAGATCGGATTC-3’ |  |

*All the primers were uesed to detected rhLZ mice and pigs in our previous studies[24,26].
