# Supplementary material for: Production of Transgenic-Cloned Pigs Expressing Large Quantities of Recombinant Human Lysozyme in Milk
Source: PLoS One. 2015 May 8;10(5):e0123551. doi: 10.1371/journal.pone.0123551 (PMC4425539; doi:10.1371/journal.pone.0123551)
Supplement: S2 Table — Values are averages ± standard deviations. Some samples at particular time were not collected, and here we use “-” to indicate. (DOCX) [file pone.0123551.s002.docx]

**S2 Table. Expression level of rhLZ in the milk of transgenic pigs (mg/L) and rhLZ enzymatic activities (U/μL) of milk collected on day 7.**

| Time | 133 | 137 | 132 | 138 | 303 | 315 | 299 | 301 | 312 | 313 |
| --- | --- | --- | --- | --- | --- | --- | --- | --- | --- | --- |
| 3 h | 342.0 ± 0.8 | 504.2 ± 53.2 | 1431.2 ± 124.9 | 1083.6 ± 47.5 | 389.4 ± 45.0 | 670.5 ± 14.0 | 1076.6 ± 40.1 | 1224.3 ± 123.0 | 983.3 ± 40.1 | 704.4 ± 15.3 |
| 6 h | 352.1 ± 1.5 | 553.1 ± 77.9 | 1404.2 ± 110.1 | 1241.7 ± 63.6 | 197.6 ± 7.8 | 706.2 ± 121.3 | 1191.8 ± 45.6 | 1349.0 ± 156.8 | 1312.8 ± 12.3 | 786.5 ± 64.7 |
| 9 h | 397.7 ± 7.0 | 488.0 ± 22.7 | 1522.2 ± 81.9 | 1314.0 ± 72.1 | 393.6 ± 8.5 | 990 ± 1.7 | 1224.9 ± 109.9 | 1181.9 ± 10.0 | 1341.6 ± 86.1 | 879.8 ± 43.1 |
| 12 h | 547.7 ± 22.7 | 514.1 ± 2.3 | 1294.1 ± 106.3 | 1447.5 ± 38.2 | 440.0 ± 32.0 | 810.3 ± 5.9 | 1064.1 ± 66.6 | 1255.2 ± 176.5 | 1336.2 ± 76.4 | 1310.3 ± 52.8 |
| 24 h | 372.2 ± 26.1 | 605.0 ± 99.1 | 1762.4 ± 34.2 | 1550.4 ± 73.4 | 578.6 ± 13.4 | 1433.3 ± 72.8 | 1466.3 ± 43.1 | 1357.7 ± 29.5 | 1354.1 ± 53.7 | - |
| 48 h | 428.3 ± 43.5 | 686.1 ± 64.5 | 2006.1 ± 37.3 | 2376.2 ± 2.3 | - | 1904.7 ± 129.0 | 2002.7 ± 91.9 | 1932.6 ± 87.4 | - | 705.0 ± 80.6 |
| 7 d | 586.8 ± 91.6 | 475.1 ± 5.3 | 1958.3 ± 97.4 | 1758.8 ± 125.4 | 784.7 ± 35.4 | 1449.5 ± 100.3 | 1330.4 ± 102.9 | 1266.0 ± 7.6 | 1779.6 ± 66.6 | - |
| 14 d | 673.8 ± 11.9 | 877.7 ± 52.8 | - | 2759.6 ± 265.0 | 831.3 ± 44.5 | 1518.3 ± 39.9 | 1541.3 ± 149.1 | 1368.2 ± 53.2 | 1971.0 ± 209.2 | 1631.6 ± 139.8 |
| 21 d | 588.3 ± 1.7 | 600.8 ± 30.8 | - | 2542.5 ± 189.2 | 691.4 ± 36.3 | 1652.6 ± 108.4 | 2010.8 ± 232.7 | 2057.3 ± 154.2 | 2292.5 ± 45.6 | 1464.5 ± 6.6 |
| 28 d | 795.5 ± 35.0 | 700.8 ± 78.1 | - | 2448.0 ± 92.1 | 666.2 ± 55.4 | 1706.6 ± 77.0 | 1596.5 ± 293.8 | 1920.8 ± 266.2 | 1642.4 ± 86.8 | 1387.7 ± 149.1 |
| Average | 508.4 ± 153.9 | 600.5 ± 125.4 | 1625.5 ± 283.3 | 1852.2 ± 618.8 | 552.5 ± 210.7 | 1284.2 ± 450.3 | 1450.5 ± 344.8 | 1491.3 ± 337.7 | 1557.0 ± 402.2 | 1108.7 ± 378.2 |
| enzymatic activities | 246.0 ± 44.2 | - | 1311.0 ± 508.6 | 877.2 ± 88.4 | 709.2 ± 87.1 | 1205.4 ± 437.8 | - | 1192.8 ± 298.8 | 1308.6 ± 518.9 | - |

Values are averages ±standard deviations. Some samples at particular time were not collected, and here we use “-”to indicate.
