# Supplementary material for: Production of Transgenic-Cloned Pigs Expressing Large Quantities of Recombinant Human Lysozyme in Milk
Source: PLoS One. 2015 May 8;10(5):e0123551. doi: 10.1371/journal.pone.0123551 (PMC4425539; doi:10.1371/journal.pone.0123551)
Supplement: S3 Table — Values are averages ± standard deviations. (DOCX) [file pone.0123551.s003.docx]

**S3 Table. The influence of rhLZ transgenic milk on the growth of *Escherichia coli* K88**

| Time | 100 µg/ml | WT | *P*-value | 50 µg/ml | WT | *P*-value |
| --- | --- | --- | --- | --- | --- | --- |
| 3h | 0.0097±0.0019 | 0.0117±0.0010 | 0.3291 | 0.0029±0.0018 | 0.0074±0.0027 | 0.1786 |
| 4h | 0.0143±0.0029 | 0.0232±0.0054 | 0.0853 | 0.0124±0.0025 | 0.0129±0.0001 | 0.7361 |
| 5h | 0.0861±0.0017 | 0.1435±0.0028 | 0.0004 | 0.0822±0.0129 | 0.1108±0.0124 | 0.1869 |
| 6h | 1.0220±0.0956 | 1.3210±0.0335 | 0.0146 | 1.018±0.0581 | 1.206±0.0653 | 0.1045 |
| 7h | 1.7220±0.0222 | 1.8040±0.0425 | 0.1555 | 1.7440±0.0523 | 1.8220±0.0554 | 0.3243 |
| 8h | 1.8600±0.0511 | 1.9390±0.0754 | 0.3590 | 1.9220±0.0560 | 1.9910±0.0402 | 0.3422 |

Values are averages ±standard deviations.
